# Study on the Role of Gastrointestinal Parasite in Irritable Bowel Syndrome Patients in a Tribal Region of India

**DOI:** 10.7759/cureus.26091

**Published:** 2022-06-19

**Authors:** Bhupati Bhusan Das, Abinash Kumar Panda, Mahadev Prasad Patra, Kedarnath Nayak

**Affiliations:** 1 Department of General Surgery, Saheed Laxman Nayak Medical College and Hospital, Koraput, IND; 2 Department of Pediatric Surgery, SCB Medical College & Hospital, Cuttack, IND; 3 Department of General Surgery, Pandit Raghunath Murmu Medical College and Hospital, Baripada, IND; 4 Department of General Surgery, Bhima Bhoi Medical College & Hospital, Bolangir, IND; 5 Department of General Surgery, Government Medical College and Hospital, Sundergarh, IND

**Keywords:** post infectectious ibs, giardia lamblia, protozoa and helminthes, entamoeba histolytica, irritable bowel syndrome

## Abstract

Background

Irritable bowel syndrome (IBS) is a functional gastrointestinal (GI) disorder in which abdominal pain is associated with a change in bowel habits. Gut inflammation might be one of the mechanisms of pathogenesis. However, the cause of IBS is not clearly understood. Post-infectious IBS (PI-IBS) is the onset of IBS after an episode of infectious gastroenteritis. While the exact pathophysiology of PI-IBS is not established, the mechanism might be an altered serotonin signaling activity, inflammation, malabsorption, and small intestinal bacterial overgrowth. Various parasites such as *Blastocystis hominis* and *Dientamoeba fragilis* have a possible role in the etiology of IBS. *Entamoeba histolytica *is one of the predominant GI parasites in developing regions of the world, and the symptoms of non-dysenteric amebic colitis may mimic those of IBS, which makes them difficult to distinguish from each other. Our study will address the relationship between the different gastrointestinal protozoan parasites in IBS and the role of antiparasitic therapy in PI-IBS. This study also aimed to determine the prevalence of GI protozoan parasites in patients with IBS in a tribal region of India.

Methods

We conducted a descriptive facility-based cross-sectional study of patients presenting with IBS to Saheed Laxman Nayak Medical College and Hospital, Koraput, Odisha, from 2017 to 2021. We collected stool samples for histopathological analysis using direct wet mount and formal-ether concentration microscopy techniques if diarrhea persisted beyond the antidiarrheal therapy. The samples from IBS patients were compared against 80 healthy control patient stool samples. We used IBM SPSS Statistics for Windows, Version 24.0 (IBM Corp., Armonk, NY,) to analyze the data.

Results

Our study included 120 patients with IBS, of whom 67 (56%) were infected with GI parasites. In the control group, 16 (20%) were infected with GI parasites, which was significantly fewer than the test group (p<0.001).

Conclusion

We found a widespread infestation with GI parasites in patients with diarrhea-predominate IBS. A parasitological stool test should be included in the diagnostic approach to IBS. Initiating early diagnosis and treatment can reduce the chance of post-infectious IBS.

## Introduction

Irritable bowel syndrome (IBS) can be defined as a functional gastrointestinal (GI) disorder in which abdominal pain is associated with a change in bowel habits [1,2]. Gut inflammation might be one of the mechanisms of pathogenesis. However, the cause of IBS is not clearly understood. A diagnosis is often made based on symptoms, and IBS often develops after a severe bout of diarrhea. There are several types of IBS: IBS-C is constipation predominant, IBS-D is diarrhea predominant, IBS-M is mixed bowel habits, and IBS-A alternates between constipation and diarrhea [3]. IBS-D causes abdominal pain and frequent urges to go to the toilet. There may be colonic cramps and splashing sounds. The stool during the attack may or may not have mucus. In IBS-M/IBS-A, the stools are hard or watery. An individual suffering from IBS will experience symptoms such as belly discomfort, pain, and trouble with bowel movement (e.g., constipation or diarrhea). A flare-up may also be possible.

Post-infectious IBS (PI-IBS) is the onset of IBS after an episode of infectious gastroenteritis [4,5], and it occurs in 4% to 31% of cases [6,7]. PI-IBS occurs primarily as traveler’s diarrhea or gastroenteritis outbreaks, and in these scenarios, IBS-D symptoms prevail. Intestinal parasites are categorized as protozoa or helminths. Protozoa include *Giardia lamblia*, *Entamoeba histolytica*, *Balantidium coli*, and *Cryptosporidium parvum*. Helminths include flatworms and nematodes [8]. Intestinal parasites are associated with three important environmental factors: contaminated drinking water, inadequate sanitation, and poor nutrition.

While the exact pathophysiology of PI-IBS is not very well established, the mechanism might be an altered serotonin signaling activity, inflammation, malabsorption, and small intestinal bacterial overgrowth [9-12]. However, Hanevik et al. mentioned that protozoan parasites, such as *Blastocystis hominis* and *Dientamoeba fragilis*, have a possible role in the etiology of IBS [13]. *Entamoeba histolytica* is one of the predominant GI parasites in developing regions of the world, and the symptoms of non-dysenteric amebic colitis may mimic those of IBS, which makes them difficult to distinguish from each other [13]. Various studies suggested that *E. histolytica* may also play a role in IBS [14,15]. Therefore, we conducted this study to determine the prevalence of different GI protozoan parasites in IBS in a tribal population in India.

## Materials and methods

We conducted a descriptive facility-based cross-sectional qualitative study of patients with age group 18 to 60 years, both male and female, presenting with symptoms suggestive of IBS in Saheed Laxman Nayak Medical College and Hospital, Koraput, Odisha, a tribal area of eastern India, from November 2017 to November 2021. All patients of the study group received a course of antidiarrheal therapy that included antibiotic, pre-probiotic, and other supportive agents for at least one month. Patients who did not improve after antidiarrheal therapy and had persistent symptoms of IBS, such as abdominal discomfort, diarrhea, and constipation, were included in the study. The study excluded patients with celiac disease, those with inflammatory bowel disease, those with HIV, and those on immunosuppression. We collected stool samples for histopathological analysis if diarrhea persisted for four weeks beyond the discontinuation of antidiarrheal therapy. We used direct smear examination and the formal ether concentration technique to detect and identify GI parasites. The samples from IBS patients were compared against 80 healthy control patient stool samples. All patients provided written informed consent. We used IBM SPSS Statistics for Windows, Version 24.0. (IBM Corp., Armonk, NY) to analyze the data. We considered p<0.05 as statistically significant.

## Results

A total of 120 IBS patients were included in the study, of whom 67 (56%) were infected with GI parasites. Of the 80 control group patients, only 16 (20%) were infected with GI parasites (p<0.001). The overall prevalence of infection among IBS and control patients was 42% (Table 1).

**Table 1:** Prevalence of GI parasites in IBS patients and control patients GI, gastrointestinal; IBS, irritable bowel syndrome

Patients	Examined, n	Positive, n (%)	p-Value
IBS	120	67 (56%)	0.0001
Control	80	16 (20%)	
Total	200	73 (42%)	

The prevalence among male and female patients in the IBS group was similar (53% and 56%, respectively); however, this difference was statistically significant (p=0.004). The prevalence of intestinal parasites among male and female patients in the control group was 22% and 17%, respectively (Table 2).

**Table 2:** Prevalence of GI parasites in IBS patients and control patients by sex GI, gastrointestinal; IBS, irritable bowel syndrome

Group	Sex	Examined, n	Positive, n (%)	p-Value
IBS patients	Male	66	35 (53%)	0.004
	Female	54	30 (56%)	
	Total	120	65 (54%)	
Control group	Male	48	10 (22%)	0.1
	Female	32	5 (17%)	
	Total	80	15 (19%)	

For the IBS patients, the prevalence of GI parasite infection was high among patients aged 31 to 40 years (66%) and those aged 21 to 30 years (61%). The lowest prevalence occurred in the age group of 10 to 20 years (14%; p=0.051). In the control group, the highest prevalence was recorded among the age group of 21 to 20 years, while the lowest prevalence was in patients older than 40 years (Table 3; p=0.03).

**Table 3:** Prevalence of GI parasites in IBS patients and control patients by age group GI, gastrointestinal; IBS, irritable bowel syndrome

Group	Age, years	Examined, n	Positive, n (%)	p-Value
IBS patients	10-20	7	1 (14%)	0.051
	21-30	38	23 (61%)	
	31-40	61	40 (66%)	
	Over 40	14	6 (43%)	
	Total	120	70 (58%)	
Control group	10-20	0	0 (%)	0.03
	21-30	25	6 (24%)	
	31-40	45	9 (20%)	
	Over 40	10	1 (10%)	
	Total	80	16 (20%)	

*Giardia lamblia* was the most common parasite present in 27.5% of IBS cases. *Entamoeba histolytica* was present in 13% of IBS cases, *Hymenolepis nana* was found in 6% of IBS cases, *Entamoeba coli* in 3%, and *Taenia* spp. and *Ascaris lumbricoides* in 2.5% each in IBS group (Table 4; p=0.004). GI parasites in the control group were less prevalent. In the control population, *G. lamblia* was present in only 10% of cases, *E. coli* in 5%, *E. histolytica* in 2.5%, *H. nana* in 1%, and *Taenia* spp. in 1% of the cases (p=0.11).

**Table 4:** Prevalence of GI parasites in IBS (n=120) and control patients (n=80) GI, gastrointestinal; IBS, irritable bowel syndrome

GI parasites	IBS group positive, n (%)	p-Value	Control group positive, n (%)	p-Value
Entamoeba histolytica	16 (13%)	0.004	2 (2.5%)	0.1
Ascaris lumbricoides	3 (2.5%)		0 (0%)	
Taenia spp.	3 (2.5%)		1 (1%)	
Hymenolepis nana	7 (6%)		1 (1%)	
Entamoeba coli	4 (3%)		4 (5%)	
Giardia lamblia	33 (27.5%)		8 (10%)	

## Discussion

We conducted this study to determine the prevalence of GI protozoan parasites in patients with IBS in a tribal region of India. Among the patients with IBS, the overall prevalence rate of a GI parasite was extremely high (56%). Eisa’s thesis study at Alzaim Alazhari University of Keryab Village residents found a prevalence similar to ours (56.6%) (Unpublished Data. M.Sc. Thesis: Eisa IM. The Efficiency of Different Technique [sic] in the Detection of Intestinal Parasites in School Children in Keryab Village, Khartoum State. Alzaim Alazhari University; 2005). However, Awole et al. reported a lower rate of 34.4% in their study in Ethiopia (34.4%) [15]. Bundy et al.’s study in Juba (Southern Sudan) found a higher rate (66%) than ours [16]. Develoux et al.’s study in 1986 in Niger found a slightly higher prevalence of GI parasitic infection than our population (57.5%) [17]. Given the statistically significantly higher infection prevalence in IBS patients with GI parasitic infections than controls, GI parasites might play an active role in the pathogenesis of IBS.

The association between IBS and GI parasites is noted in previous studies from non-Asian countries. The clinical manifestations of *G. lamblia* infection vary from asymptomatic diarrhea to acute and chronic diarrhea with abdominal pain. Hanevik et al. reported that *G. lamblia* infection might cause functional bowel disease, including IBS [13]. In a study from Norway, 66 (81%) of 82 patients with a *G. lamblia* infection had symptoms of functional bowel diseases, and IBS was the most common subtype (47%) [18]. In non-Asian countries, the incidence of G. lamblia infection is more common in travelers returning from giardiasis-endemic areas (i.e., the frequency of giardiasis is 5.3 in 100,000 people) [19]. Taylor et al. reported that approximately 80% of patients contracting *G. lamblia* might develop symptoms of IBS [14]. The association of GI parasites with IBS was further supported by Chaudhary and Truelove [19] in their study of *E. histolytica* in IBS patients [19]. However, Morgan et al.’s study in Nicaragua negates the association between intestinal parasites and IBS, concluding that residents of a low-income country are exposed to these microbes early in life and develop an immune tolerance [20].

The study conducted in tribal populations appears biased as such populations are already known to be a high prevalence cohort for gut infestations. IBS is seeing an upward trend in rural and tribal populations, which may be due to environmental and dietary changes. A further comparative study in the urban population is thus suggested. Our study had another important limitation in its small sample size. Future studies with larger sample sizes, including people from a wider geographical area, would mitigate this limitation and further elucidate the role of GI parasites in the pathogenesis of PI-IBS.

## Conclusions

This study aimed to determine the prevalence of GI protozoan parasites in IBS in a tribal population in India. We found a significantly higher prevalence of GI parasites in IBS patients than in the control group. The presence of *G. lamblia* and *E. histolytica* in the stool sample of IBS patients seems to indicate an association between the GI parasite and the pathogenesis of IBS. Diagnostic evaluations should include stool sample microscopic examinations for IBS patients in endemic areas. Early diagnosis and treatment can reduce the chance of PI-IBS development.
